# 3-(2*H*-Benzotriazol-2-yl)-1-(4-fluoro­phen­yl)propan-1-one

**DOI:** 10.1107/S1600536810013917

**Published:** 2010-04-21

**Authors:** Zhi-Fang Pan

**Affiliations:** a Weifang Medical University, Weifang 261042, People’s Republic of China

## Abstract

In the title compound, C_15_H_12_FN_3_O, the benzotriazole ring system is essentially planar, with a maximum deviation from the least-squares plane of 0.016 (3) Å. The dihedral angle between this ring system and the fluoro-substituted benzene ring is 67.97 (2)°. The crystal structure is stabilized by weak inter­molecular C—H⋯N inter­actions.

## Related literature

For applications of benzotriazole derivatives, see: Chen & Wu (2005 ▶). For standard bond distances, see: Allen *et al.* (1987 ▶).
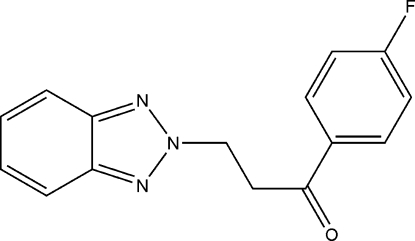


## Experimental

### 

#### Crystal data

C_15_H_12_FN_3_O
*M* *_r_* = 269.28
Monoclinic, 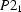

*a* = 5.7858 (12) Å
*b* = 5.6814 (11) Å
*c* = 19.313 (4) Å
β = 90.77 (3)°
*V* = 634.8 (2) Å^3^
*Z* = 2
Mo *K*α radiation
μ = 0.10 mm^−1^
*T* = 293 K
0.20 × 0.18 × 0.10 mm

#### Data collection

Bruker SMART CCD diffractometer
3943 measured reflections
1240 independent reflections
1122 reflections with *I* > 2σ(*I*)
*R*_int_ = 0.135

#### Refinement

*R*[*F* ^2^ > 2σ(*F* ^2^)] = 0.081
*wR*(*F* ^2^) = 0.210
*S* = 1.07
1240 reflections
181 parameters
1 restraint
H-atom parameters constrained
Δρ_max_ = 0.30 e Å^−3^
Δρ_min_ = −0.34 e Å^−3^

### 

Data collection: *SMART* (Bruker, 1997 ▶); cell refinement: *SAINT* (Bruker, 1997 ▶); data reduction: *SAINT*; program(s) used to solve structure: *SHELXS97* (Sheldrick, 2008 ▶); program(s) used to refine structure: *SHELXL97* (Sheldrick, 2008 ▶); molecular graphics: *SHELXTL* (Sheldrick, 2008 ▶); software used to prepare material for publication: *SHELXTL*.

## Supplementary Material

Crystal structure: contains datablocks global, I. DOI: 10.1107/S1600536810013917/lh5027sup1.cif

Structure factors: contains datablocks I. DOI: 10.1107/S1600536810013917/lh5027Isup2.hkl

Additional supplementary materials:  crystallographic information; 3D view; checkCIF report

## Figures and Tables

**Table 1:** Hydrogen-bond geometry (Å, °)

*D*—H⋯*A*	*D*—H	H⋯*A*	*D*⋯*A*	*D*—H⋯*A*
C14—H14*B*⋯N1^i^	0.97	2.58	3.511 (3)	161

Symmetry code: (i) 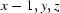.

**Fig. 1.**
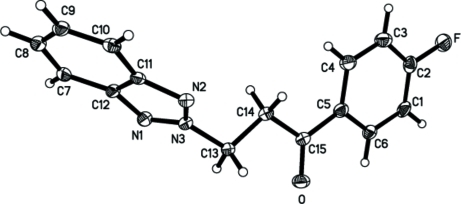
The molecular structure of (I), drawn with 30% probability ellipsoids.

**Fig. 2.**
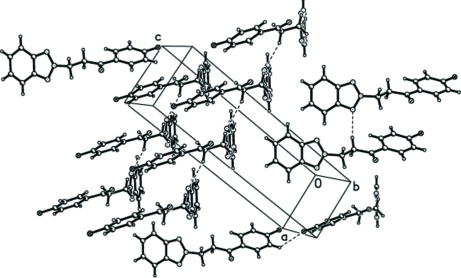
Part of the crystal structure of (I) showing hydrogen bonds as dashed lines.

## supplementary crystallographic information

### Comment

1*H*-Benzotriazole and its derivatives are an important class of compounds because they exhibit a broad spectrum of pharmacological activities such as antifungal, antitumor and antineoplastic activities (Chen & Wu., 2005). 1*H* and 2*H*-Benzotriazole are tautomers. We report here the synthesis and structure of the title compound, (I) (Fig. 1), as part of our ongoing studies on new benzotriazole compounds with potential bioactivity. All bond lengths (Allen *et al.*, 1987) and angles in (I) are within normal ranges. The benzotriazole ring system is essentially planar with a maximum deviation from the least squares plane of 0.016 (3)Å. The dihedral angle between this ring system and the fluro substituted benzene ring is 67.97 (2). The crystal structure is stabilized by weak intermolecular C—H···N interactions.

### Experimental

To a solution of 1-(4-ethylphenyl)-3-(dimethylamino)propan-1-one (12.05 g, 0.05 mol) in water (25 ml) was added benzotriazole (7.1 g, 0.06 mol). The mixture was heated under reflux for 5 h. The solution was filtered,concentrated and purified by flash chromatography (silica gel,using petroleum ether-ethylacetate(4:1 *v*/*v*). to afford the title compound. Colourless single crystals suitable for X-ray diffraction study were obtained by slow evaporation of a ethanol solution over a period of 5 d.

### Refinement

In the absence of significant anomalous dispersion effects the Friedel pairs were merged. All H atoms were located in difference Fourier maps and constrained to ride on their parent atoms, with C—H distances in the range 0.93–0.97 Å, and with *U*_iso_(H) = 1.2 *U*_eq_(C) .

### Crystal data

**Table:**

C_15_H_12_FN_3_O	*F*(000) = 280
*M**_r_* = 269.28	*D*_x_ = 1.409 Mg m^−^^3^
Monoclinic, *P*2_1_	Mo *K*α radiation, λ = 0.71073 Å
Hall symbol: P 2yb	Cell parameters from 1874 reflections
*a* = 5.7858 (12) Å	θ = 1.1–25.0°
*b* = 5.6814 (11) Å	µ = 0.10 mm^−^^1^
*c* = 19.313 (4) Å	*T* = 293 K
β = 90.77 (3)°	Block, colorless
*V* = 634.8 (2) Å^3^	0.20 × 0.18 × 0.10 mm
*Z* = 2	

### Data collection

**Table:**

Bruker SMART CCD diffractometer	1122 reflections with *I* > 2σ(*I*)
Radiation source: fine-focus sealed tube	*R*_int_ = 0.135
graphite	θ_max_ = 25.0°, θ_min_ = 1.1°
φ and ω scans	*h* = −6→6
3943 measured reflections	*k* = −6→6
1240 independent reflections	*l* = −22→20

### Refinement

**Table:**

Refinement on *F*^2^	Primary atom site location: structure-invariant direct methods
Least-squares matrix: full	Secondary atom site location: difference Fourier map
*R*[*F*^2^ > 2σ(*F*^2^)] = 0.081	Hydrogen site location: inferred from neighbouring sites
*wR*(*F*^2^) = 0.210	H-atom parameters constrained
*S* = 1.07	*w* = 1/[σ^2^(*F*_o_^2^) + (0.1432*P*)^2^ + 0.1388*P*] where *P* = (*F*_o_^2^ + 2*F*_c_^2^)/3
1240 reflections	(Δ/σ)_max_ = 0.002
181 parameters	Δρ_max_ = 0.30 e Å^−^^3^
1 restraint	Δρ_min_ = −0.34 e Å^−^^3^

### Special details

**Table:**

Geometry. All e.s.d.'s (except the e.s.d. in the dihedral angle between two l.s. planes) are estimated using the full covariance matrix. The cell e.s.d.'s are taken into account individually in the estimation of e.s.d.'s in distances, angles and torsion angles; correlations between e.s.d.'s in cell parameters are only used when they are defined by crystal symmetry. An approximate (isotropic) treatment of cell e.s.d.'s is used for estimating e.s.d.'s involving l.s. planes.
Refinement. Refinement of F^2^ against ALL reflections. The weighted R-factor wR and goodness of fit S are based on F^2^, conventional R-factors R are based on F, with F set to zero for negative F^2^. The threshold expression of F^2^ > 2sigma(F^2^) is used only for calculating R-factors(gt) etc. and is not relevant to the choice of reflections for refinement. R-factors based on F^2^ are statistically about twice as large as those based on F, and R- factors based on ALL data will be even larger.

### Fractional atomic coordinates and isotropic or equivalent isotropic displacement parameters (Å^2^)

**Table:**

	*x*	*y*	*z*	*U*_iso_*/*U*_eq_	
F	0.0574 (7)	1.0117 (7)	0.01386 (19)	0.0505 (11)	
O	0.9115 (8)	1.3049 (7)	0.1980 (2)	0.0434 (12)	
N1	1.3671 (7)	0.6871 (8)	0.3093 (2)	0.0278 (10)	
N2	1.0658 (8)	0.6958 (8)	0.3810 (2)	0.0300 (10)	
N3	1.1720 (8)	0.7892 (8)	0.3293 (2)	0.0265 (10)	
C9	1.3499 (10)	0.1759 (10)	0.4572 (3)	0.0316 (13)	
H9A	1.3402	0.0609	0.4913	0.038*	
C15	0.8110 (10)	1.1189 (10)	0.1966 (3)	0.0300 (13)	
C14	0.8827 (10)	0.9205 (9)	0.2440 (3)	0.0292 (12)	
H14A	0.9270	0.7856	0.2164	0.035*	
H14B	0.7524	0.8746	0.2720	0.035*	
C12	1.3905 (9)	0.5068 (10)	0.3556 (3)	0.0274 (12)	
C5	0.6140 (11)	1.0790 (10)	0.1461 (3)	0.0326 (13)	
C13	1.0823 (9)	0.9904 (10)	0.2907 (3)	0.0293 (12)	
H13A	1.2047	1.0571	0.2630	0.035*	
H13B	1.0315	1.1103	0.3229	0.035*	
C11	1.2016 (9)	0.5119 (10)	0.4001 (3)	0.0276 (12)	
C2	0.2439 (10)	1.0338 (10)	0.0570 (3)	0.0344 (14)	
C6	0.5609 (10)	1.2550 (10)	0.0984 (3)	0.0315 (13)	
H6A	0.6528	1.3891	0.0966	0.038*	
C10	1.1818 (10)	0.3418 (10)	0.4533 (3)	0.0285 (12)	
H10A	1.0595	0.3438	0.4841	0.034*	
C8	1.5400 (10)	0.1698 (10)	0.4116 (3)	0.0336 (13)	
H8A	1.6498	0.0512	0.4164	0.040*	
C3	0.2883 (12)	0.8551 (12)	0.1038 (3)	0.0416 (15)	
H3A	0.1945	0.7223	0.1050	0.050*	
C4	0.4757 (11)	0.8775 (10)	0.1490 (3)	0.0356 (14)	
H4A	0.5090	0.7594	0.1809	0.043*	
C7	1.5654 (10)	0.3346 (10)	0.3607 (3)	0.0309 (12)	
H7A	1.6910	0.3332	0.3311	0.037*	
C1	0.3754 (11)	1.2338 (10)	0.0540 (3)	0.0376 (15)	
H1B	0.3395	1.3526	0.0226	0.045*	

### Atomic displacement parameters (Å^2^)

**Table:**

	*U*^11^	*U*^22^	*U*^33^	*U*^12^	*U*^13^	*U*^23^
F	0.048 (2)	0.046 (2)	0.057 (2)	0.0034 (19)	−0.0271 (17)	−0.0036 (19)
O	0.048 (3)	0.026 (2)	0.056 (3)	−0.007 (2)	−0.019 (2)	0.006 (2)
N1	0.027 (2)	0.025 (2)	0.031 (3)	0.001 (2)	−0.0019 (17)	0.0008 (19)
N2	0.027 (2)	0.021 (2)	0.042 (3)	−0.001 (2)	−0.0053 (18)	−0.002 (2)
N3	0.028 (2)	0.020 (2)	0.032 (3)	0.0000 (18)	−0.0043 (18)	−0.0002 (19)
C9	0.040 (3)	0.025 (3)	0.029 (3)	−0.010 (3)	−0.012 (2)	0.006 (2)
C15	0.030 (3)	0.022 (3)	0.037 (3)	0.001 (2)	−0.004 (2)	−0.004 (2)
C14	0.033 (3)	0.023 (3)	0.032 (3)	0.001 (2)	0.000 (2)	−0.002 (2)
C12	0.030 (3)	0.020 (2)	0.032 (3)	−0.002 (2)	−0.010 (2)	0.004 (2)
C5	0.038 (3)	0.025 (3)	0.035 (3)	0.002 (2)	−0.004 (2)	−0.005 (2)
C13	0.028 (3)	0.020 (2)	0.040 (3)	0.000 (2)	−0.011 (2)	−0.001 (2)
C11	0.028 (3)	0.021 (2)	0.034 (3)	−0.011 (2)	−0.009 (2)	0.002 (2)
C2	0.032 (3)	0.039 (3)	0.031 (3)	0.011 (3)	−0.008 (2)	−0.007 (3)
C6	0.035 (3)	0.026 (3)	0.034 (3)	0.004 (2)	−0.005 (2)	0.006 (2)
C10	0.032 (3)	0.027 (3)	0.026 (3)	−0.008 (2)	−0.0030 (19)	−0.001 (2)
C8	0.036 (3)	0.027 (3)	0.038 (3)	−0.004 (2)	−0.012 (2)	0.001 (3)
C3	0.044 (4)	0.030 (3)	0.050 (4)	−0.007 (3)	−0.010 (3)	−0.002 (3)
C4	0.042 (3)	0.030 (3)	0.035 (3)	−0.003 (3)	−0.007 (2)	−0.001 (3)
C7	0.028 (3)	0.024 (3)	0.040 (3)	0.000 (2)	−0.009 (2)	−0.006 (2)
C1	0.043 (4)	0.025 (3)	0.044 (4)	0.010 (3)	−0.010 (3)	0.009 (2)

### Geometric parameters (Å, °)

**Table:**

F—C2	1.360 (7)	C5—C6	1.390 (8)
O—C15	1.207 (7)	C5—C4	1.399 (9)
N1—N3	1.331 (7)	C13—H13A	0.9700
N1—C12	1.364 (7)	C13—H13B	0.9700
N2—N3	1.293 (7)	C11—C10	1.416 (8)
N2—C11	1.356 (7)	C2—C1	1.369 (9)
N3—C13	1.457 (7)	C2—C3	1.381 (9)
C9—C10	1.356 (8)	C6—C1	1.370 (9)
C9—C8	1.419 (9)	C6—H6A	0.9300
C9—H9A	0.9300	C10—H10A	0.9300
C15—C14	1.506 (7)	C8—C7	1.367 (9)
C15—C5	1.508 (7)	C8—H8A	0.9300
C14—C13	1.510 (7)	C3—C4	1.388 (9)
C14—H14A	0.9700	C3—H3A	0.9300
C14—H14B	0.9700	C4—H4A	0.9300
C12—C11	1.400 (8)	C7—H7A	0.9300
C12—C7	1.410 (8)	C1—H1B	0.9300
			
N3—N1—C12	102.4 (4)	H13A—C13—H13B	108.0
N3—N2—C11	104.3 (5)	N2—C11—C12	107.7 (5)
N2—N3—N1	117.3 (4)	N2—C11—C10	132.1 (5)
N2—N3—C13	123.2 (5)	C12—C11—C10	120.2 (5)
N1—N3—C13	119.4 (5)	F—C2—C1	119.2 (5)
C10—C9—C8	122.9 (5)	F—C2—C3	118.2 (5)
C10—C9—H9A	118.5	C1—C2—C3	122.6 (5)
C8—C9—H9A	118.5	C1—C6—C5	121.0 (5)
O—C15—C14	120.9 (5)	C1—C6—H6A	119.5
O—C15—C5	120.4 (5)	C5—C6—H6A	119.5
C14—C15—C5	118.7 (5)	C9—C10—C11	116.7 (5)
C15—C14—C13	111.6 (5)	C9—C10—H10A	121.6
C15—C14—H14A	109.3	C11—C10—H10A	121.6
C13—C14—H14A	109.3	C7—C8—C9	121.5 (5)
C15—C14—H14B	109.3	C7—C8—H8A	119.3
C13—C14—H14B	109.3	C9—C8—H8A	119.3
H14A—C14—H14B	108.0	C4—C3—C2	118.7 (6)
N1—C12—C11	108.4 (5)	C4—C3—H3A	120.6
N1—C12—C7	129.2 (5)	C2—C3—H3A	120.6
C11—C12—C7	122.4 (5)	C3—C4—C5	119.5 (6)
C6—C5—C4	119.6 (5)	C3—C4—H4A	120.2
C6—C5—C15	118.6 (5)	C5—C4—H4A	120.2
C4—C5—C15	121.7 (5)	C8—C7—C12	116.2 (6)
N3—C13—C14	111.3 (5)	C8—C7—H7A	121.9
N3—C13—H13A	109.4	C12—C7—H7A	121.9
C14—C13—H13A	109.4	C2—C1—C6	118.6 (5)
N3—C13—H13B	109.4	C2—C1—H1B	120.7
C14—C13—H13B	109.4	C6—C1—H1B	120.7

### Hydrogen-bond geometry (Å, °)

**Table:**

*D*—H···*A*	*D*—H	H···*A*	*D*···*A*	*D*—H···*A*
C14—H14B···N1^i^	0.97	2.58	3.511 (3)	161

Symmetry codes: (i) *x*−1, *y*, *z*.
